# Real-World Prevalence, Treatment Patterns, and Economic Impact of EGFR- and ALK-Targeted Therapies in Non-Small Cell Lung Cancer: A Nationwide Analysis from Greece

**DOI:** 10.3390/curroncol32100542

**Published:** 2025-09-27

**Authors:** George Gourzoulidis, Catherine Kastanioti, George Mavridoglou, Theodore Kotsilieris, Anastasios Tsolakidis, Konstantinos Mathioudakis, Dikaios Voudigaris, Charalampos Tzanetakos

**Affiliations:** 1 Department of Business and Organizations Administration, University of the Peloponnese, 24100 Kalamata, Greece; a.kastanioti@uop.gr (C.K.); t.kotsilieris@uop.gr (T.K.); 2 Department of Accounting and Finance, School of Management, University of the Peloponnese, 24100 Kalamata, Greece; ge.mavridoglou@go.uop.gr; 3 IDIKA SA—E-Government Center for Social Security Services, 10551 Athens, Greece; tsolakidis@idika.gr (A.T.); mathioudakis@idika.gr (K.M.); 4 Health Through Evidence GP, 17456 Athens, Greece; d.voudigaris@hte.gr (D.V.); c.tzanetakos@hte.gr (C.T.)

**Keywords:** lung cancer, drug utilization patterns, nationwide real-world evidence, Greece

## Abstract

Objectives: To determine the prescribing prevalence of epidermal growth factor receptor (EGFR)- and anaplastic lymphoma kinase (ALK)-positive non-small cell lung cancer (NSCLC) patients in Greece and examine patterns of first-line tyrosine kinase inhibitor (TKI) utilization and associated treatment costs using nationwide real-world data. Methods: A retrospective analysis of the national e-prescription database was performed, identifying patients initiating first-line treatment (FLT) for EGFR- or ALK-positive NSCLC between 1 January 2020 and 31 December 2022. Demographic characteristics, prescribing prevalence data, drug utilization patterns, total annual drug expenditures, and per patient treatment costs were assessed. All statistical analyses were performed using the statistical software SPSS-v.29. Results: Overall, 1188 EGFR-positive (mean age of 70.93 ± 11.6) and 246 (mean age of 64.26 ± 12.6) ALK-positive NSCLC patients initiated FLT during the three-year study period. EGFR mutations were slightly more common in females (53%), peaking in the 70–79 age group (35%). ALK mutations were also more common among females (52%), particularly within the 60–79 age group. In EGFR-positive patients, osimertinib usage markedly increased from 41% in 2020 to 63% in 2022, primarily displacing afatinib (from 32% to 22%) and erlotinib (from 24% to 14%), with gefitinib prescriptions falling below 2%. Among ALK-positive patients, crizotinib utilization declined significantly from 60% to 16%, whereas alectinib increased to 59% by 2022. Annual EGFR-related total drug expenditures remained stable (€11.5 million in 2020 vs. €11.9 million in 2022), driven primarily by increasing osimertinib usage. Similarly, ALK-related annual drug expenditures showed stability, with costs predominantly attributed to rising alectinib utilization. Conclusions: This nationwide analysis highlights the rapid adoption of second- and third-generation TKIs for EGFR- and ALK-positive NSCLC in Greece, reflecting evolving clinical practice patterns. Although the target patient populations are relatively small, the associated economic burden is considerable. To ensure long-term sustainability of the Greek healthcare system, policymakers should critically assess the cost-effectiveness of these innovative therapies and align resource allocation with value-based care principles.

## Simple Summary

Lung cancer is the leading cause of cancer-related deaths in Greece and worldwide. In recent years, new treatments called tyrosine kinase inhibitors (TKIs) have been developed to target specific genetic mutations in lung cancer, such as EGFR and ALK. These medicines often work better and have fewer side effects than traditional chemotherapy. To understand how these treatments are used in Greece, we analyzed nationwide prescription data from 2020 to 2022. We looked at all patients who began treatment with an EGFR- or ALK-targeted drug during this period. Our study found that Greek clinical experts quickly switched from older TKIs to newer ones, such as osimertinib and alectinib, which are now the most commonly prescribed. Although the number of treated patients was relatively small, the cost to the national health system was still significant. However, total spending remained stable over time. This was largely due to national pricing regulations and negotiated discounts, which helped control costs despite the use of newer and more expensive drugs. These findings provide valuable insights for health authorities and policymakers, helping them ensure fair and sustainable access to innovative cancer treatments for patients in Greece.

## 1. Introduction

Non-small cell lung cancer (NSCLC) represents approximately 85% of all lung cancer diagnoses and remains one of the leading causes of cancer-related mortality worldwide [1]. Significant advancements in precision medicine have reshaped treatment paradigms, particularly through the identification and targeting of specific genetic alterations such as epidermal growth factor receptor (EGFR) mutations and anaplastic lymphoma kinase (ALK) rearrangements [2,3,4]. Clinical guidelines, including those from the European society for medical oncology (ESMO) and Hellenic society of medical oncology (HeSMO) now recommend routine testing for these biomarkers in advanced NSCLC due to the demonstrated efficacy and improved patient outcomes associated with targeted therapies [5,6,7].

EGFR- and ALK-targeted therapies, including tyrosine kinase inhibitors (TKIs), have significantly altered the treatment landscape by offering superior progression-free survival, improved quality of life, and reduced toxicity compared to traditional chemotherapy [8,9]. Despite these clinical advantages, the widespread adoption and utilization patterns of these targeted therapies vary significantly across countries due to differences in healthcare infrastructure, clinical practices, health policies, and economic constraints [10,11].

In Greece, lung cancer poses a substantial clinical and economic burden. It is the leading cause of cancer-related death [1], accounting for significant healthcare resource utilization and costs, including hospitalizations, medications, diagnostic procedures, and indirect societal costs due to lost productivity [12].

Access to innovative therapies such as TKIs in Greece follows a structured reimbursement pathway. After approval by the European medicines agency (EMA), the manufacturer must submit a health technology assessment (HTA) dossier to the national HTA Committee. This evaluation considers factors such as clinical effectiveness, safety, cost-effectiveness, and budget impact compared to existing treatment options [13].

If the HTA outcome is positive, the therapy proceeds to formal price negotiations with the Negotiation Committee of the National Organization for Health Services Provision (EOPYY), the national payer [13]. These negotiations determine confidential discounts or managed entry agreements, which are applied on top of existing statutory rebates.

Once an agreement is reached, the Ministry of Health includes the medicine in the official catalogue of reimbursable medicines, making it available for routine clinical use through the national electronic prescription platform (IDIKA).

However, the lack of national evidence limits stakeholders’ ability to assess the affordability, sustainability, and value of precision oncology in clinical practice. In particular, the budgetary impact of rapidly evolving TKI therapies remains underexplored in the Greek setting, where cost-containment policies and health technology assessments are becoming increasingly central to reimbursement decisions.

Thus, the current study aims to address these gaps by presenting a nationwide real-world analysis of EGFR- and ALK-targeted therapy use among patients with NSCLC in Greece. Specifically, we assess the prescribing prevalence, patterns of treatment uptake and the direct economic impact of these therapies within the context of the Greek National Health System. By providing comprehensive and locally relevant data, this study seeks to inform evidence-based decision-making in clinical practice and policy formulation and support the development of sustainable access strategies for innovative cancer therapies in Greece.

## 2. Materials and Methods

### 2.1. Study Design and Data Source

This study was a retrospective observational analysis utilizing anonymized data derived from the nationwide electronic prescription database of Greece, managed by e-Government Center for Social Security Services (IDIKA S.A.) The database covers nearly the entire Greek population by capturing prescriptions reimbursed by the National Organization for Health Services Provision (EOPYY), which operates under the authority of the Greek Ministry of Health [14,15]. The e-prescription system is fully integrated at the national level, covering both public and private healthcare sectors, and is mandatory for nearly all prescriptions, particularly those reimbursed through national health insurance. It systematically records data on all medications prescribed by physicians and dispensed at payer pharmacies across Greece, thus providing a comprehensive and reliable source of real-world data on medication utilization.

Patients were identified based on the prescription of first-line TKIs specifically approved for EGFR- or ALK-positive NSCLC between 1 January 2020 and 31 December 2022. Biomarker status was inferred based on drug indication, as molecular test results are not included in the e-prescription database. Anonymized data were utilized in this study following formal authorization by the administration of IDIKA S.A. and approval by the data protection officer (DPO) of the Hellenic Ministry of Health. Moreover, the institutional review board of the University of the Peloponnese approved the study protocol. As the analysis was based exclusively on secondary data extracted from a national health administrative database, individual informed consent was not required. The study was conducted in full compliance with national regulations governing the protection of personal data and adhered to the ethical principles outlined in the declaration of Helsinki and its subsequent amendments.

### 2.2. Study Cohort, Outcomes and Definitions

Patients with NSCLC who initiated treatment with at least one TKI between 1 January 2020 and 31 December 2022, were included in the analysis. Eligible cases were identified through the nationwide electronic prescription database using International Classification of Diseases, Tenth Revision (ICD-10) diagnostic codes of: C34, C34.0, C34.1, C34.2, C34.3, C34.8 and C34.9 (Table S1 in the Supplementary Material). These diagnostic entries were cross-referenced with prescriptions for EGFR- or ALK-targeted TKIs, identified by their Anatomical Therapeutic Chemical (ATC) classification codes: Gefitinib (L01EB01), Erlotinib (L01EB02), Afatinib (L01EB03), Osimertinib (L01EB04), Dacomitinib (L01EB07), Crizotinib (L01ED01), Ceritinib (L01ED02), Alectinib (L01ED03), Brigatinib (L01ED04), and Lorlatinib (L01ED05).

Only patients who initiated first-line TKI therapy within the defined study window were included to ensure consistency in treatment initiation. The index date was defined as the date of the first executed prescription for a TKI. Patients remained in the same treatment line until a documented switch to another TKI or a transition to chemotherapy occurred. Hence, the treatment time per patient was defined as the difference between the date of the first and last recorded dispensation of an ALK or EGFR TKI in the period from 1 January 2020 to 31 December 2022.

Data extracted from the national prescription database were stratified into two primary cohorts based on molecular subtype: patients receiving EGFR TKIs and those receiving ALK TKIs. For each patient, demographic variables including age and sex were recorded. Prescription patterns were evaluated in terms of the frequency and distribution of specific EGFR- and ALK-targeted agents.

To assess the economic burden associated with EGFR- and ALK-targeted therapies, the annual pharmaceutical expenditure for TKIs was calculated based on reimbursed drug unit prices applicable for each calendar year within the study period. Following the payer perspective, the drug cost estimations were derived from the ex-factory prices published in the official price bulletin of the Greek Ministry of Health [16], which represent the only publicly available pricing data. In accordance with local legislation, the ex-factory price was first reduced by 8.74% to determine the hospital procurement price. This amount was subsequently discounted by an additional 5% to calculate the final invoiced price used for reimbursement calculations. Using these adjusted prices, annual treatment cost was estimated for the entire study cohort, as well as separately for the EGFR-positive and ALK-positive NSCLC sub-populations. Confidential rebates and clawbacks negotiated between the Ministry of Health and pharmaceutical companies were not publicly available and thus not included in this analysis.

In addition, the prevalence of patients receiving at least one EGFR or ALK TKI prescription was estimated using population data from the Hellenic Statistical Authority as the denominator. Age and sex-standardized prevalence estimates were calculated separately for each molecular subgroup, and a weighted average prevalence was reported across the full observation period (2020–2022). The demographic distribution of EGFR and ALK NSCLC, including age-specific and sex-specific prevalence rates, was also analyzed.

### 2.3. Statistical Analysis

Categorical variables were reported using frequencies (n) and percentages (%), while continuous variables were described using means and standard deviations (SD). Before analysis, continuous variables were assessed for normality using the Shapiro–Wilk test.

Prevalence estimates were calculated at the overall population level and further stratified by sex and age, using decade-based age groups for the adult Greek population. No statistical inference or imputations of missing data were performed. Prevalence rates per 100,000 population were calculated using age- and sex-stratified census data from the Hellenic Statistical Authority (ELSTAT) as denominators. Ninety-five percent confidence intervals (CIs) were estimated, and subgroup comparisons were performed with chi-square tests. Given the descriptive and exploratory nature of the analysis, no adjustment for multiple testing was applied. Comparisons for continuous variables were performed using independent samples *t*-tests for normally distributed continuous variables, and chi-square tests were used for categorical variables. Statistical significance was defined as a *p*-value < 0.05. All statistical analyses were conducted using the statistical software package IBM SPSS Statistics for Windows, Version 29.

## 3. Results

### 3.1. Patient Characteristics and Prescribing Prevalence of TKI Based Treatment

**Figure 1:**
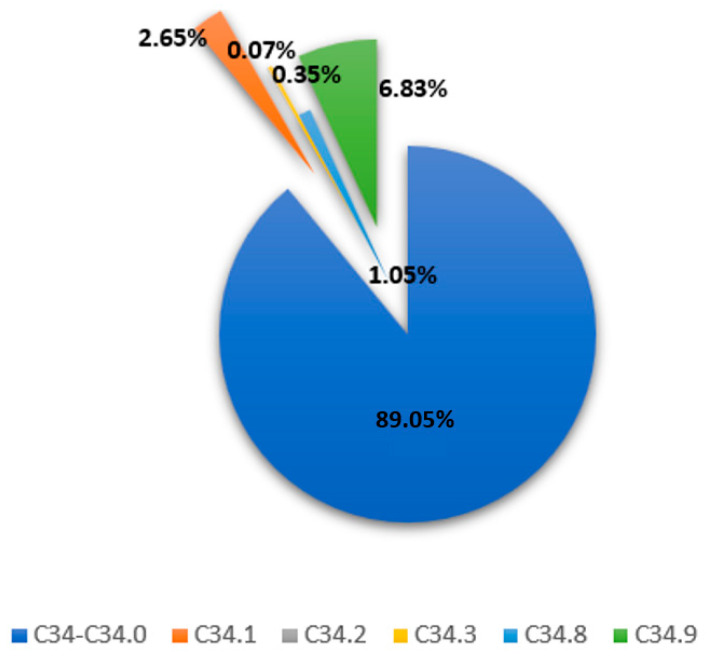
Distribution of EGFR/ALK patients per ICD-10.

According to the nationwide prescription database, a total of 1188 patients with EGFR-positive and 246 with ALK-positive NSCLC initiated first-line therapy with at least one TKI during the three-year study period. (Table S1 in the Supplementary Material for ICD-10 codes and Figure S1 for the patient-flow diagram). The mean (SD) age of patients in the EGFR-positive cohort was 70.93 years (±11.16), while ALK-positive patients were younger, with a mean age of 64.26 years (±12.6). In terms of diagnostic classification, the majority of patients (approximately 89%) with at least one prescription were recorded under the ICD-10 code C34 and C34.0. This was followed by C34.9, which accounted for 6.83% of cases. The distribution of the most frequently recorded ICD-10 codes is presented in Figure 1.

Among EGFR-positive patients, 53% were females with the highest frequency reported in the 70–79 age group (36%). Prescribing prevalence remained relatively high in those aged 80 years and above (27%), although slightly lower than the 70–79 peak (Table 1). In the ALK-positive cohort, females also comprised the majority (52%), with the highest prescribing prevalence observed in the 60–69 (26%) and 70–79 (28%) age groups (Table 2).

The three-year period prescribing prevalence of EGFR-positive NSCLC was estimated at 10.09 per 100,000 males (95% CI: 9.25–10.92) and 13.99 per 100,000 females (95% CI: 12.89–15.07), yielding an overall prevalence of 11.84 per 100,000 population (95% CI: 11.16–12.51). For ALK-positive patients, the corresponding prescribing prevalence was 2.15 per 100,000 in males (95% CI: 1.77–2.54) and 2.81 per 100,000 in females (95% CI: 2.32–3.30), with an overall prevalence of 2.45 per 100,000 (95% CI: 2.14–2.76) (Table 1).

Sex-stratified analyses across age groups for EGFR-positive patients revealed no statistically significant differences in prevalence between males and females (all *p*-values > 0.05). While females consistently exhibited slightly higher prescribing prevalence rates in most age bands particularly in the 50–59 and 60–69 groups, none of these differences reached statistical significance. The largest numerical difference was noted in the 50–59 age group, yet with a *p*-value of 0.16, it did not attain significance, suggesting no strong evidence of sex predominance in EGFR-mutant NSCLC prevalence (Table 1).

In contrast, for ALK-positive patients, most age groups did not demonstrate statistically significant sex-based differences. However, in the 70–79 age group, males exhibited a significantly higher prescribing prevalence than females (8.99 vs. 5.18 per 100,000; *p* = 0.023), indicating a notable male predominance within this subgroup (Table 2). In all other age groups, sex differences were not statistically meaningful (*p* > 0.05), supporting the conclusion that sex-related disparity in ALK-positive NSCLC prevalence is limited to the 70–79 age category (Table 2).

### 3.2. Treatment Utilization Patterns

**Figure 2:**
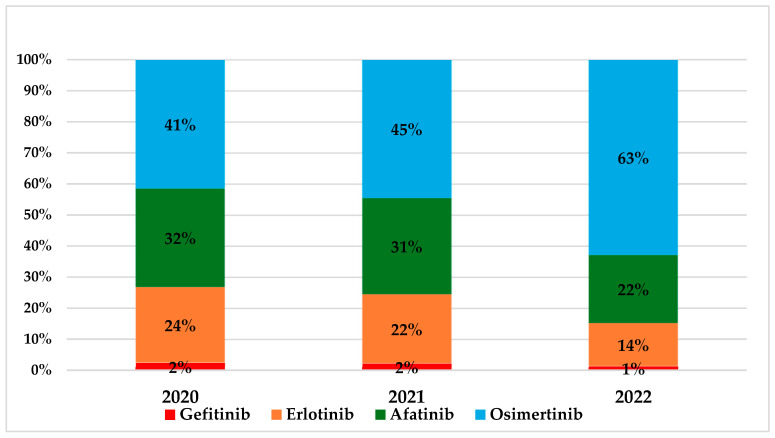
Distribution of the EGFR tyrosine kinase inhibitor treatment per year. EGFR: epidermal growth factor receptor. Note: Percentages may not total 100% due to rounding.

Among patients with EGFR-mutant NSCLC, there was a clear and progressive shift in treatment patterns over the three-year study period, reflecting the evolving landscape of targeted therapy. Osimertinib, which accounted for 41% of first-line EGFR TKI use in 2020, rose to 45% in 2021 and reached 63% in 2022 (Figure 2), becoming the most commonly prescribed agent across all age groups and both sexes.

In parallel, the use of earlier-generation TKIs showed consistent declines. Afatinib, initially prescribed to 130 patients in 2020 (32%), dropped to 125 in 2021 and further declined to 82 (22%) in 2022. Erlotinib use similarly decreased from 24% in 2020 to 14% by 2022 (Figure 2). Gefitinib prescriptions were nearly phased out over the study period, falling below 2% by 2022, consistent with declining clinical preference and guideline revisions (Figure 2). It should be noted that no patients were found to have initiated first-line therapy with the second-generation EGFR TKI dacomitinib during the observation period. This finding is consistent with the fact that dacomitinib had not yet received reimbursement approval in Greece at the time of the study [17].

Table 3 provides a detailed breakdown of patient’s distributions stratified by age group, sex, and year. Osimertinib was prescribed across all age groups, but its uptake was particularly pronounced in the 60–69 and 70–79 age groups. In 2022 alone, 61 patients aged 60 and above (30 males, 31 females aged 60–69; 37 males, 33 females aged 70–79) initiated first-line therapy with osimertinib, supporting its growing role in elderly populations.

Afatinib maintained a consistent role across 2020 and 2021, especially in the 60–79 age range but was increasingly replaced by osimertinib in 2022. Erlotinib showed moderate use in patients aged 60–79 in the first two years, but its use diminished by the third year, with only 20 male and 32 female patients initiating treatment in 2022.

Sex-stratified data revealed relatively balanced prescribing patterns between male and female patients across all EGFR TKIs. EGFR TKI treatment selection was not associated with sex (*p* = 0.83) or age group (*p* = 0.51), indicating consistent prescribing across demographic subgroups. These findings suggest that the uptake of EGFR-targeted therapies trends were broadly consistent across sexes with minor differences which may reflect subtle variations in physician preferences or patient-specific clinical profiles (Table 3).

**Figure 3:**
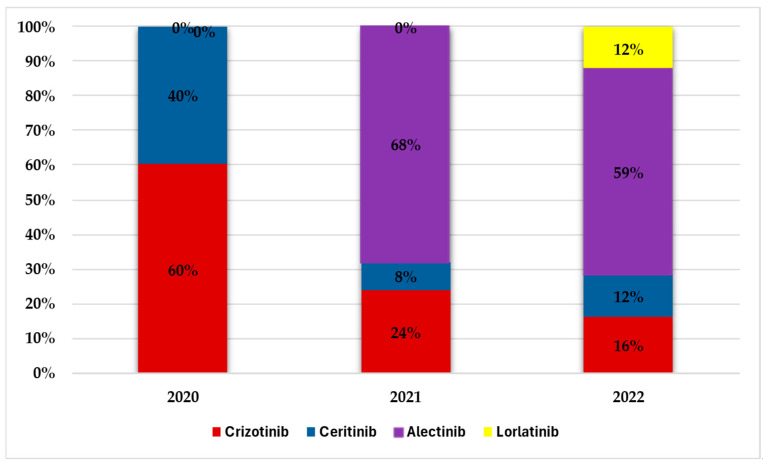
Distribution of the ALK tyrosine kinase inhibitor treatment per year. ALK: anaplastic lymphoma kinase. Note: Percentages may not total 100% due to rounding.

Treatment patterns for ALK-positive NSCLC patients evolved considerably over the study period, reflecting a clear shift toward the adoption of second-generation TKIs. In 2020, crizotinib was the most commonly prescribed agent, accounting for 60% of ALK TKI use, followed by ceritinib at 40%. Alectinib was not prescribed in 2020 indicating its limited availability or absence from reimbursement at that time, and lorlatinib had not yet entered clinical practice for this indication (Figure 3).

By 2021, there was a striking transition in clinical practice. Alectinib emerged as the leading therapy, prescribed to 57 patients (27 males, 30 females), comprising 68% of ALK TKI use (Table 4). Crizotinib use dropped sharply to 24% (12 males, 8 females), while ceritinib accounted for only 8% of prescriptions (Figure 3).

In 2022, alectinib maintained its dominant position, accounting for 59% of prescriptions. Notably, lorlatinib entered clinical practice with a modest uptake (12%), suggesting its growing role in sequential treatment. Ceritinib continued to decline, constituting only 12% of prescriptions, and crizotinib dropped further to 16% (Figure 3).

The transition toward alectinib was recorded across all age groups, particularly among patients aged 60–79 reflecting clinical preferences favoring its efficacy and CNS activity (Table 4). Lorlatinib was prescribed most frequently in older age groups, especially those aged 50 and older likely to reflect its use as a second-line option following disease progression on earlier-generation TKIs.

Sex-stratified data again revealed balanced utilization patterns across ALK TKIs, though alectinib showed slightly higher uptake among females in the 70–79 and 80+ age groups. Ceritinib, by contrast, was more frequently prescribed to males in 2020, but this difference did not persist in subsequent years (Table 4). Statistical analysis confirmed that there was no significant association between patient sex and the type of ALK TKI prescribed (*p* = 0.414), suggesting that ALK-targeted therapies were distributed equitably between male and female patients throughout the study period. In contrast, a statistically significant association was reported between age group and the ALK TKI prescribed (*p* = 0.003), indicating that age may have influenced therapeutic selection.

Although brigatinib received EMA approval for use in the first-line setting on 6 April 2020, no patients were noted to have initiated first-line therapy with brigatinib during the study period. As a result, due to the time required for national HTA, reimbursement negotiations, and price approval procedures under Greek pharmaceutical legislation, brigatinib had not yet been integrated into the national electronic prescribing system at the time of this study. Consequently, it was not captured in the observed utilization patterns.

### 3.3. Cost of First Line Tyrosine Kinase Inhibitor Treatment

Annual expenditures for EGFR-targeted TKIs remained relatively stable throughout the study period, rising only marginally from €11.49 million in 2020 to €11.88 million in 2022 (Table 5). This budgetary stability conceals a marked shift in the composition of spending: by 2022, osimertinib alone accounted for the vast majority of EGFR-related pharmaceutical expenditures, increasing from €9.32 million in 2020 to €10.73 million. In contrast, expenditures for earlier-generation agents declined substantially, with erlotinib costs decreasing from €734,907 to €316,867, and afatinib costs dropping from €1.30 million to €775,012 over the same period (Table 5). These changes reflect evolving clinical practice patterns favoring third-generation EGFR TKIs and confirm that osimertinib has become the dominant cost driver within this therapeutic class.

As for the mean cost per patient per year for EGFR TKIs showed only a modest increase, from €28,024 in 2020 to €31,758 in 2022 (Table 5). This reflects the gradual replacement of lower-cost agents such as gefitinib and erlotinib with newer, higher-cost treatments, especially osimertinib, yet without imposing a substantial increase in total expenditure.

A similar trend was observed for ALK-targeted therapies. Total annual expenditure remained stable, ranging from €3.45 million in 2020 to €3.30 million in 2022. However, the treatment landscape changed significantly. Crizotinib, the dominant cost driver in 2020 (€2.08 million), saw a sharp decline in utilization and cost (€418,496 in 2022), while alectinib expenditures increased substantially, peaking at €2.71 million in 2021 and remaining high at €2.52 million in 2022. Lorlatinib entered clinical use in 2022 with a limited budget impact (€85,105), reflecting its early adoption and restricted use (Table 5).

At the patient level, the mean cost per ALK-positive patient rose from €39,220 in 2020 to €44,609 in 2022. This increase can be primarily attributed to the growing uptake of alectinib, which had the highest per-patient annual cost among ALK TKIs in 2022, averaging €47,614 (Table 5). These data reinforce the dual role of alectinib as both a clinically preferred and economically influential agent in the treatment of ALK-positive NSCLC.

It is important to note that, although newer-generation TKIs such as osimertinib and alectinib are associated with higher acquisition costs, overall, the total drug spending did not increase significantly during the study period. This relative stability may, in part, be explained by the legislated reductions in drug prices applied annually in Greece, including mandatory statutory discounts on ex-factory prices and regulated pricing mechanisms. As a result, even as prescribing shifted toward more expensive agents, annual per-patient cost increases remained moderate.

## 4. Discussion

This is, to our knowledge, the first nationwide study in Greece to examine real-world prescribing patterns, TKI utilization, and the prevalence of patients with NSCLC harboring EGFR mutations or ALK rearrangements during the period 2020–2022.

Our findings highlight that the mean age of patients in the EGFR-positive cohort was 70.9 years, while ALK-positive patients were younger, with a mean age of 64.2 years. These findings are broadly consistent with other real-world studies, where EGFR mutations are more frequently observed in older females and ALK rearrangements tend to occur in younger patients. The age distribution in our cohort closely mirrors those reported in other similar real-world studies [18,19,20,21].

Although minor numerical differences in TKI uptake were observed across sexes, statistical analysis revealed no significant association between sex and first-line TKI selection in either cohort. In contrast, TKI selection was significantly associated with age in the ALK-positive cohort, reflecting greater uptake of newer agents such as alectinib among older patients.

In addition, our study reveals a decisive and rapid shift toward next-generation TKIs. For EGFR-positive NSCLC, osimertinib emerged as the dominant first-line agent by 2022, replacing earlier generation TKIs. For ALK-positive, alectinib overtook crizotinib within a year of its introduction, reflecting both alignment with updated ESMO and HeSMO guidelines [5,6,7] and supported by evidence of superior efficacy and more favorable toxicity profiles [9,22,23]. Moreover, similar prescribing patterns have been reported in other real-world studies, further supporting the consistency and generalizability of these findings [18,20].

From an economic perspective, the transition toward next-generation TKIs has not yet driven a substantial increase in total national expenditure. Stable overall spending, despite the higher acquisition costs of osimertinib and alectinib, likely reflects statutory price reductions, competitive market effects, and Greece’s regulated reimbursement framework. Nevertheless, per-patient costs remain high, especially for ALK-positive disease, underscoring the need for careful financial stewardship.

However, it is important to acknowledge that the study period did not fully capture the availability and uptake of all relevant first-line therapies options. Agents such as lorlatinib, brigatinib, and dacomitinib received their EMA approvals for first line use after 2020. As a result, due to the time required for HTA, reimbursement negotiations, and national price approvals as mandated by Greek pharmaceutical legislation these therapies were not yet integrated into the national electronic prescribing system during the observation period of this study. Notably, by the time of writing, lorlatinib and brigatinib have completed the necessary HTA and reimbursement procedures and are now reimbursed by the Greek national payer. In contrast, dacomitinib remains unreimbursed in Greece until now [17].

These findings carry important implications for oncology policy. First, the rapid uptake of innovative TKIs demonstrates the responsiveness of the reimbursement system but also underscores the importance of integrating real-world evidence into HTA processes. Prevalence and utilization data, such as those provided in present study, are essential for cost-effectiveness and budget impact studies and can also inform national strategies to optimize access to innovative therapies.

Second, beyond access, long-term sustainability requires value-based reimbursement approaches, including outcome-based agreements that link payment to demonstrated benefit. The importance of economic evaluations within HTA processes should be underscored to ensure that decisions reflect not only drug acquisition costs but also the broader value these therapies deliver to the healthcare system. Despite their importance, such studies in lung cancer remain scarce in the Greek setting, with only limited published evidence currently available [24,25]. It is also important to recognize that while these targeted therapies improve outcomes by extending the time patients live without disease progression and staying longer on treatment they also lead to longer treatment durations and higher costs per patient. In addition to treatment advances, diagnostic innovations such as next-generation sequencing panels are transforming clinical practice [26]. These tests allow multiple genetic mutations to be identified at once, improving diagnostic speed and precision. However, they come with higher upfront costs for the healthcare system. As both treatment and diagnostic strategies evolve, these trends have important financial and policy implications. They must be carefully considered in HTA and resource planning, in order to ensure that innovation can be integrated equitably and sustainably within the national health system.

Additionally, the observed demographic trends emphasize the need for more targeted lung cancer screening and molecular testing strategies. Investment in diagnostic infrastructure and expanded access to comprehensive molecular profiling are essential to fully realize the clinical potential of targeted therapies [27]. Such efforts would not only enable more personalized treatment selection but also promote more efficient allocation of public healthcare resources, ultimately delivering greater clinical benefit to patients with lung cancer [28,29].

This study has several limitations. First, the use of prescription data, while nationally representative, does not capture clinical outcomes, adverse events, or patient-specific clinical decisions. Second, the analysis included only patients who obtained their medications via the IDIKA national electronic prescription system, which may have led to a slight underestimation of TKI use in the target populations. Nevertheless, the IDIKA national electronic prescription system provides a robust source of real-world data on medication use, with near complete coverage of the Greek population. This extensive scope enhances the reliability and validity of pharmacological utilization data. Notably, IDIKA data have previously been used to evaluate treatment patterns and medication use in other disease areas, including diabetes mellitus [14], osteoporosis [30], rheumatic disorders [15], and multiple sclerosis [31], further supporting its applicability in real-world research.

Third, pharmaceutical expenditures were estimated based on official ex-factory prices minus statutory discounts, consistent with Greek legislation. However, additional confidential rebates and clawbacks between the Ministry of Health and pharmaceutical companies are not publicly disclosed and therefore could not be incorporated. As a result, our estimates may slightly overstate the actual net financial burden for the payer, although these undisclosed adjustments are unlikely to alter the relative trends reported. Additionally, per-patient treatment costs were assessed within fixed calendar-year windows (2020–2022). As in similar real-world studies utilizing administrative or claims databases, this approach may slightly underestimate treatment exposure and associated costs for patients who initiated therapy late in the calendar year. Fourth, the study reflects prescribing trends from 2020 through 2022; therefore, it does not capture more recent changes in clinical practice or the introduction of additional targeted therapies. Nonetheless, Greece has lacked publicly available data on the number of patients with ALK or EGFR-positive NSCLC, as well as on treatment patterns in these molecular subgroups. The absence of a national lung cancer registry in Greece significantly limits the ability to validate prevalence estimates, monitor therapeutic trends, and conduct robust population-based analyses. Establishing such a registry should be a national priority to enhance oncology research and inform evidence-based policymaking.

Moreover, the study period (2020–2022) coincided with the COVID-19 pandemic, which likely disrupted diagnostic and treatment pathways, particularly among older adults who constituted the majority of the study population. These systemic pressures may partially account for the de-cline in newly treated NSCLC cases observed over time. Notably, lung cancer was one of the most common malignancies among patients with cancer and concurrent COVID-19, and individuals with lung cancer were particularly susceptible to infection, likely due to pre-existing abnormalities in the respiratory epithelium that facilitate viral entry into pulmonary tissue [32].

Lastly, while the IDIKA national e-prescription database provides detailed information on prescribing patterns, it does not link molecular diagnostic registries or contain data on specific EGFR mutation subtypes or ALK rearrangement variants. As a result, we were unable to determine whether all patients with NSCLC underwent biomarker testing, or whether testing rates changed over time during the study period.

## 5. Conclusions

This nationwide real-world analysis provides the first assessment of the prevalence, utilization patterns, and economic burden of EGFR- and ALK-targeted therapies in Greece. The study findings highlight the rapid uptake of second and third-generation TKIs for EGFR- and ALK-positive NSCLC in Greece, reflecting evolving clinical practice patterns. Although the target patient populations are relatively small, the associated economic burden is considerable. Moreover, study results underscore the need for targeted strategies to optimize molecular testing, improve access to precision therapies, and promote responsible stewardship of healthcare resources. To support the long-term sustainability of the Greek healthcare system, it is essential that reimbursement and policy decisions be guided by evidence on therapeutic value and affordability and the principles of value-based care. Additionally, this study underscores broader systemic challenges, including persistent gaps in data infrastructure and the absence of a national lung cancer registry. Addressing these limitations is critical to enabling more informed oncology policymaking, supporting transparent pricing negotiations, and ensuring timely patient access to innovative treatments in the era of personalized medicine.

## Figures and Tables

**Table 1:** Three-year period prescribing prevalence of EGFR-positive NSCLC patients according to age group and sex.

Age Groups	Number of EGFR-Positive NSCLC Patients			Prevalence of the EGFR-Positive NSCLC Patients in the Greek Population			*p*-Value
	Male	Females	Total	Prevalence (/100,000) Male 95% CI	Prevalence (/100,000) Females 95% CI	Prevalence (/100,000) Total 95% CI	
18–39	6	2	8	0.23 (0.05–0.42)	0.16 (−0.06–0.39)	0.21 (0.06–0.36)	0.659
40–49	21	26	47	2.63 (1.51–3.76)	3.26 (2.00–4.51)	2.94 (2.10–3.79)	0.467
50–59	50	68	118	6.63 (4.80–8.47)	9.50 (7.35–11.65)	8.10 (6.68–9.52)	0.161
60–69	130	160	290	20.63 (17.08–24.18)	23.04 (19.47–26.61)	21.89 (19.37–24.41)	0.349
70–79	200	215	415	43.87 (37.79–49.94)	39.81 (34.49–45.13)	41.67 (37.66–45.67)	0.323
80 and older	150	160	310	49.87 (41.30–57.03)	34.71 (29.33–40.09)	40.47 (35.96–44.97)	0.226
Total	557	631	1188	10.09 (9.25–10.92)	13.99 (12.89–15.07)	11.84 (11.16–12.51)	0.149

NSCLC: non-small cell lung cancer; EGFR: epidermal growth factor receptor, CI: confidence interval.

**Table 2:** Three-year period prescribing prevalence of ALK-positive NSCLC patients according to age and sex.

Age Groups	Number of ALK-Positive NSCLC Patients			Prevalence of the ALK-Positive NSCLC Patients in the Greek Population			*p*-Value
	Male	Females	Total	Prevalence (/100,000) Male 95% CI	Prevalence (/100,000) Females 95% CI	Prevalence (/100,000) Total 95% CI	
18–39	6	3	9	0.23 (0.05–0.42)	0.24 (−0.03–0.52)	0.24 (0.08–0.39)	0.947
40–49	11	14	25	1.38 (0.56–2.19)	1.75 (0.84–2.67)	1.57 (0.95–2.18)	0.550
50–59	20	29	49	2.65 (1.49–3.82)	3.67 (2.34–5.01)	3.17 (2.29–4.06)	0.262
60–69	30	33	63	4.76 (3.06–6.46)	4.75 (3.13–6.37)	4.76 (3.58–5.93)	0.994
70–79	41	28	69	8.99 (6.24–11.75)	5.18 (3.26–7.10)	6.93 (5.29–8.56)	0.023
80 and older	11	20	31	3.61 (1.47–5.74)	4.34 (2.44–6.24)	4.05 (2.62–5.47)	0.621
Total	119	127	246	2.15 (1.77–2.54)	2.81 (2.32–3.30)	2.45 (2.14–2.76)	0.369

NSCLC: non-small cell lung cancer; ALK: anaplastic lymphoma kinase, CI: confidence interval.

**Table 3:** Distribution of EGFR TKI patients by year stratified by age and sex.

Age Groups	Gefitinib		Erlotinib		Afatinib		Osimertinib	
2020	Male	Females	Male	Females	Male	Females	Male	Females
18–39	0	0	0	0	0	0	3	1
40–49	0	1	1	3	1	1	4	6
50–59	2	1	0	4	4	5	12	10
60–69	1	1	10	12	12	16	18	20
70–79	1	1	19	23	22	24	25	30
80 and older	1	1	12	16	22	23	20	21
**Total**	**5**	**5**	**42**	**58**	**61**	**69**	**82**	**88**
**Age Groups**	**Male**	**Females**	**Male**	**Females**	**Male**	**Females**	**Male**	**Females**
**2021**								
18–39	0	0	0	0	1	0	1	0
40–49	0	0	4	1	5	3	3	5
50–59	1	0	1	8	4	7	10	16
60–69	1	1	6	15	12	16	20	21
70–79	3	1	15	19	23	28	30	24
80 and older	1	1	9	12	12	14	27	23
**Total**	**6**	**3**	**35**	**55**	**57**	**68**	**91**	**89**
**Age Groups**	**Male**	**Females**	**Male**	**Females**	**Male**	**Females**	**Male**	**Females**
**2022**								
18–39	0	0	0	1	0	0	1	0
40–49	0	0	0	0	0	1	3	5
50–59	0	0	1	3	1	2	14	12
60–69	1	1	5	8	14	18	30	31
70–79	1	1	10	14	14	17	37	33
80 and older	1	0	4	6	7	8	34	35
**Total**	**3**	**2**	**20**	**32**	**36**	**46**	**119**	**116**

EGFR: epidermal growth factor receptor, TKI: tyrosine kinase inhibitor.

**Table 4:** Distribution of ALK TKI patients by year stratified by age and sex.

Age Groups	Crizotinib		Ceritinib		Alectinib		Lorlatinib	
2020	Male	Females	Male	Females	Male	Females	Male	Females
18–39	3	0	0	1	0	0	0	0
40–49	2	2	3	1	0	0	0	0
50–59	4	6	0	8	0	0	0	0
60–69	3	5	9	1	0	0	0	0
70–79	11	10	3	7	0	0	0	0
80 and older	4	3	0	2	0	0	0	0
**Total**	**27**	**26**	**15**	**20**	**0**	**0**	**0**	**0**
**Age Groups**	**Male**	**Females**	**Male**	**Females**	**Male**	**Females**	**Male**	**Females**
**2021**								
18–39	3	1	0	0	2	0	0	0
40–49	1	3	0	1	2	4	0	0
50–59	3	1	0	0	6	5	0	0
60–69	4	1	1	1	9	14	0	0
70–79	1	1	2	0	3	3	0	0
80 and older	0	1	1	1	5	4	0	0
**Total**	**12**	**8**	**4**	**3**	**27**	**30**	**0**	**0**
**Age Groups**	**Male**	**Females**	**Male**	**Females**	**Male**	**Females**	**Male**	**Females**
**2022**								
18–39	0	1	0	0	1	0	0	0
40–49	1	1	0	1	0	3	0	0
50–59	1	2	1	1	7	3	0	1
60–69	2	1	0	2	2	8	1	0
70–79	2	0	2	0	14	7	0	0
80 and older	0	1	0	0	0	8	0	0
**Total**	**6**	**6**	**3**	**4**	**24**	**29**	**1**	**1**

ALK: anaplastic lymphoma kinase, TKI: tyrosine kinase inhibitor.

**Table 5:** Annual and Per-Patient Cost of First-Line EGFR and ALK Tyrosine Kinase Inhibitors (2020–2022).

EGFR TKIs			
Treatment	2020	2021	2022
Gefitinib	131,329	102,228	52,818
Erlotinib	734,907	589,702	316,867
Afatinib	1,303,616	1,197,318	775,012
Osimertinib	9,320,131	8,535,158	10,732,643
**Total cost per treatment per year (€)**	**11,489,983**	**10,424,407**	**11,877,340**
**EGFR TKIs**			
**Treatment**	**2020**	**2021**	**2022**
Gefitinib	13,133	11,359	10,564
Erlotinib	7349	6552	6094
Afatinib	10,028	9579	9451
Osimertinib	54,824	47,418	45,671
**Cost per patient per year (€)**	**28,024**	**25,803**	**31,758**
**ALK TKIs**			
**Treatment**	**2020**	**2021**	**2022**
Crizotinib	2,081,949	771,661	418,496
Ceritinib	1,369,425	273,885	273,885
Alectinib	0	2,714,004	2,523,548
Lorlatinib	0	0	85,105
**Total cost per treatment per year (€)**	**3,451,374**	**3,759,550**	**3,301,034**
**ALK TKIs**			
**Treatment**	**2020**	**2021**	**2022**
Crizotinib	39,282	38,583	34,875
Ceritinib	39,126	39,126	39,126
Alectinib	0	47,614	47,614
Lorlatinib	0	0	42,552
**Cost per patient per year (€)**	**39,220**	**44,757**	**44,609**

EGFR: epidermal growth factor receptor, ALK: anaplastic lymphoma kinase, TKI: tyrosine kinase inhibitor, €: Euro.

## Data Availability

All input data for the study are available in the tables published in this manuscript.

## Supplementary Materials

The following supporting information can be downloaded at: https://www.mdpi.com/article/10.3390/curroncol32100542/s1, Table S1. Classification of Lung Cancer Diagnoses (ICD-10), Figure S1. Patient flow diagram.
