# Supplementary material for: Real-World Prevalence, Treatment Patterns, and Economic Impact of EGFR- and ALK-Targeted Therapies in Non-Small Cell Lung Cancer: A Nationwide Analysis from Greece
Source: Curr Oncol. 2025 Sep 27;32(10):542. doi: 10.3390/curroncol32100542 (PMC12563277; doi:10.3390/curroncol32100542)
Supplement: Supplementary file 1 [file curroncol-32-00542-s001.zip › curroncol-3837542-supplementary.pdf]

## Supplementary Materials

**Table S1.** Classification of Lung Cancer Diagnoses (ICD-10)

| ICD-10 | Diagnosis Description | Explanations | EGFR/ALK patients per ICD-10 |
|---|---|---|---|
| **C34** | Malignant neoplasm of bronchus and lung data | General category for primary lung cancer. | 1277 |
| **C34.0** | Main bronchus | Malignant tumor originating in the main bronchus (either left or right). | |
| **C34.1** | Upper lobe, bronchus or lung | Cancer localized in the upper lobe of the lung. | 38 |
| **C34.2** | Middle lobe, bronchus or lung | Malignancy in the middle lobe, usually of the right lung (left lung doesn't have a middle lobe). | 1 |
| **C34.3** | Lower lobe, bronchus or lung | Cancer in the lower lobe of either lung. | 5 |
| **C34.8** | Overlapping lesion of bronchus and lung | The tumor overlaps two or more contiguous sites, making it hard to localize to one specific part. | 15 |
| **C34.9** | Bronchus or lung, unspecified | Used when the specific site within the lung is unknown or not specified. | 98 |

EGFR: epidermal growth factor receptor, ALK: anaplastic lymphoma kinase

**Figure S1.** Patient flow diagram

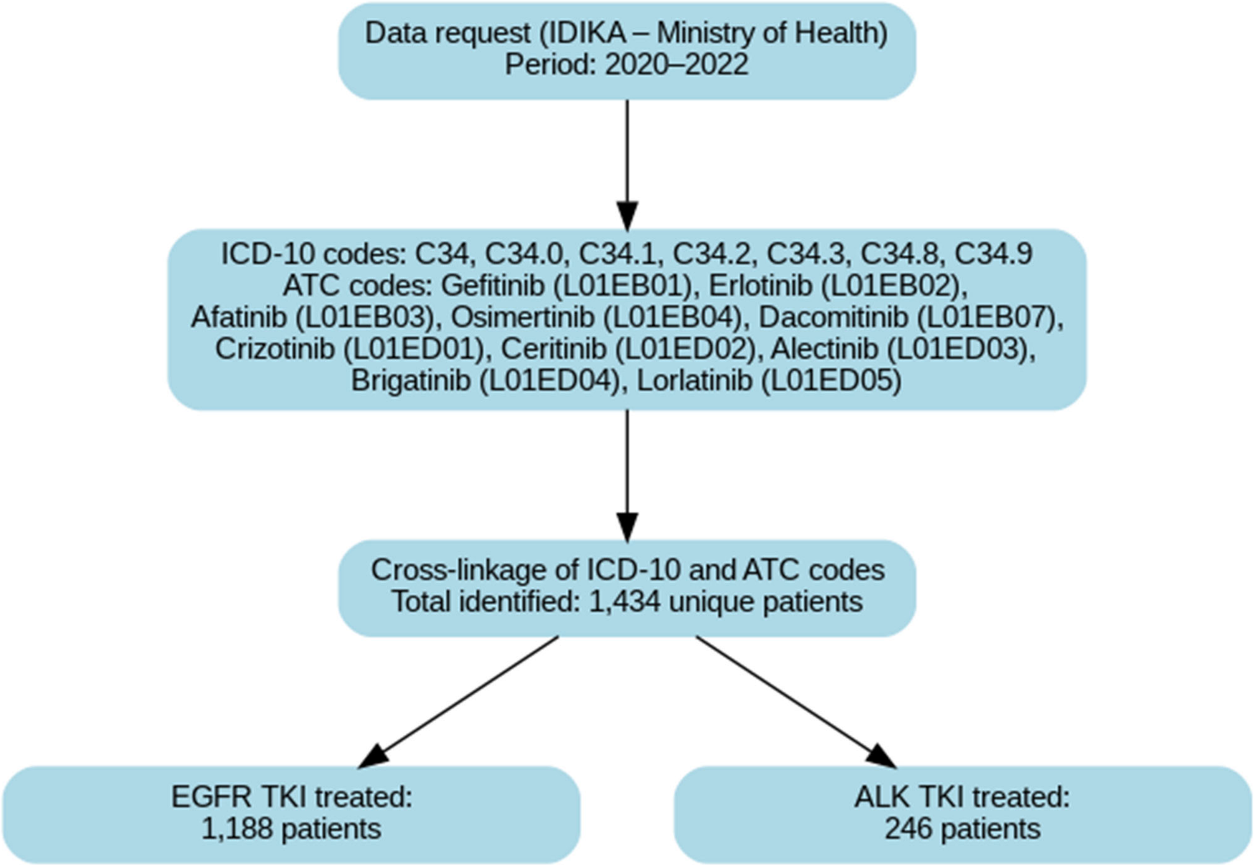


EGFR: epidermal growth factor receptor, ALK: anaplastic lymphoma kinase, ATC: Anatomical Therapeutic Chemical
